# Lifetime and Momentary Psychotic Experiences in Adult Males and Females With an Autism Spectrum Disorder

**DOI:** 10.3389/fpsyt.2020.00766

**Published:** 2020-08-03

**Authors:** Kim van der Linden, Claudia Simons, Thérèse van Amelsvoort, Machteld Marcelis

**Affiliations:** ^1^ GGzE, Mental Healthcare Institution Eindhoven, Eindhoven, Netherlands; ^2^ Department of Psychiatry and Neuropsychology, School for Mental Health and Neuroscience (MHeNS), Maastricht University, Maastricht, Netherlands

**Keywords:** autism spectrum disorder, stress, psychotic experiences, negative affect, momentary assessment

## Abstract

**Background:**

Existing research shows that adults with an autism spectrum disorder (ASD) are more vulnerable to develop overt psychosis. However, studies investigating (subclinical) psychotic experiences (PE) in ASD are scarce, and it is unknown if PE are accompanied with more distress in adults with ASD compared to the general population. This study examined lifetime PE and accompanying distress, momentary PE levels, and the impact of daily life stress and negative affect (NA) on momentary PE in males and females with ASD compared to controls.

**Methods:**

In 50 adults with ASD (males N= 26, females N= 24) and 51 adults without ASD (males N= 26, females N= 25), the Community Assessment of Psychic Experiences (CAPE) was used to analyze group differences in frequency and distress of lifetime subclinical positive, negative, and depressive symptoms. The Experience Sampling Method (ESM) was used to measure momentary PE, NA, and stress (activity-related, event-related, and social stress) for 10 days. Multilevel analyses were conducted to test whether stress and NA were associated with momentary PE and whether these associations were modified by group or sex.

**Results:**

Adults with ASD reported more lifetime CAPE negative and depressive symptoms, but similar levels of PE, than controls. Higher levels of accompanying distress were found in participants with ASD for each subscale. With respect to ESM momentary PE, higher levels were reported by adults with ASD and a stronger association between event-related stress and momentary PE was found compared to controls. This was not the case for NA, activity-related, and social stress. Overall, no significant differences between male and female outcomes were found.

**Conclusion:**

Adults with ASD are more prone to encounter lifetime subclinical negative and depressive symptoms and accompanying distress compared to adults without ASD. Similar levels of lifetime PE in both groups were still accompanied with more distress in the ASD group. Furthermore, higher levels of ESM momentary PE were found in participants with ASD. Additionally, event-related stress may act as a risk factor for PE in both females and males with ASD, with a stronger risk-increasing effect than in their control counterparts.

## Introduction

Individuals with an autism spectrum disorder (ASD) are more prone to develop overt psychosis relative to those without ASD (1, 2). General population studies have shown that psychotic experiences (PE) are an important risk factor for a psychotic disorder (3, 4), psychopathology (5, 6), and suicidal ideation (7, 8). Still, studies investigating PE in ASD are limited with an inconsistent pattern of results. For example, while two studies found significant associations between childhood autistic traits and PE in adolescence (9, 10), Taylor et al. (11) demonstrated weak or non-significant associations. Given that PE may lead to more severe psychopathology, it is essential to enhance knowledge about its (risk for) occurrence in ASD. Identifying sex differences may be necessary for understanding the underlying mechanisms of PE in ASD, which can lead to effective prevention and better-tailored treatment. Previous studies in general population samples demonstrated significantly higher levels of PE in females than in males (12, 13). However, we only found one study in children with ASD, which showed that 57% of girls with ASD experienced schizophrenia spectrum traits compared with 28% of boys (14).

Stress is a well-known risk factor in the emergence of psychosis. Individuals who have experienced childhood adversity, trauma, or adverse life events have an increased risk of developing subclinical PE (15–20) or a psychotic disorder (21–23). In the last two decades, there has been increased interest in studying the influence of minor daily stressors on momentary PE, also known as psychotic reactivity (24, 25). A widely used method to investigate psychotic reactivity is the Experience Sampling Method (ESM). The ESM is an ecological momentary assessment (EMA) tool to gather information from participants about their experiences in the context of the natural flow of daily life. Typically, multiple times a day, short questionnaires are presented to participants at semi-random moments in time over several consecutive days. This method is less susceptible to recall bias and has been applied to a wide range of psychiatric disorders (26). Although ESM research in ASD is still limited, the feasibility and usefulness of this method have been supported (27–29). Previous ESM studies observed an increased psychotic reactivity in patients at increased risk for psychosis compared with controls (30, 31). Currently, the interplay between stress and momentary PE in ASD has not yet been investigated. However, in another paper on this sample, we reported an increased negative affect (NA) in response to daily stressors in those with ASD relative to controls (submitted for publication). NA may also be directly associated with momentary PE (32–36). More specifically, a recent ESM study demonstrated that NA predicted paranoia, but, conversely, paranoia did not predict changes in NA in patients with a psychotic disorder (35). To date, no study has investigated the association between NA and momentary PE in adults with ASD.

Our first aim was to examine the frequency of experiences related to the extended psychosis phenotype and accompanying distress using the Community Assessment of Psychic Experiences (CAPE, a validated retrospective self-report questionnaire) (37, 38). That is, three individual dimensions (i.e., subclinical positive, negative, and depressive symptoms), as well as the total CAPE score, were investigated. The reason to examine beyond the positive symptom dimension is that the extended psychosis phenotype is multidimensional in nature and complements a general transdiagnostic psychosis factor (6). Our second aim was to investigate the presence and course of ESM momentary subclinical psychotic phenomena in daily life and their association with minor daily stressors and NA. Therefore, the main objectives of the current study were to examine group (ASD versus controls) and sex differences in i) levels of lifetime psychic experiences (positive, negative and depressive symptoms) and accompanying distress, ii) levels of momentary PE, iii) the impact of different types of daily stressors on momentary PE (psychotic reactivity), and iv) the impact of NA on momentary PE.

## Method

### Sample

The final sample included 50 participants with an ASD diagnosis (N= 26 males, N= 24 females) and 51 controls (N= 26 males, N= 25 females) between 18 and 65 years of age. Participants with ASD were recruited by contacting mental healthcare facilities in the South of the Netherlands, through patient associations, and *via* social media. The first author (KL) conducted the Autism Diagnostic Observation Schedule II (ADOS-2) (39) module 4 (fluent speech) in all participants of the ASD group to confirm their diagnoses. Only those participants with ASD who had i) a short-term psychological treatment history (maximum 2 years), and ii) no past psychiatric admission were included. Medication use and other psychiatric disorders were no cause for exclusion except in the case of acute psychotic symptoms, suicidal tendencies, or a bipolar disorder. The Mini-International Neuropsychiatric Interview (MINI) (40, 41) was used to assess the presence of psychiatric disorders in participants with ASD. Controls without a developmental or psychiatric disorder were recruited *via* social media. Participants were excluded if they had a first-degree family member diagnosed with, or suspected of having, ASD. The Autism Spectrum Quotient (AQ) (42, 43) was used to identify the degree of ASD features in controls; a score above 26 led to exclusion (44). The MINI was also used to exclude any control participants with a current psychiatric disorder. General exclusion criteria were i) suffering from known genetic abnormalities, brain injury, epilepsy, or metabolic disorders, and ii) an intelligence quotient (IQ) below 70. The latter was screened with two subtests (matrix reasoning and vocabulary) of the Wechsler Adult Intelligence Scale—Fourth Edition (WAIS-IV) (45). Six participants were excluded during the screening procedure (due to an IQ below 70 or ADOS-2 scores under the threshold for an ASD diagnosis). The sample characteristics are summarized in **Table 1**.

**Table 1:** Sociodemographic and clinical characteristics of the research sample.

	ASD (N = 50)	Controls (N = 51)	Group comparisons	
			Test statistic	P
Demographic Variable				
Age, mean (SD), range	41.1 (12.9), 18-64	35.5 (12.2), 18-63	F = 4.95	.028
Sex (m/f)	26/24	26/25	X^2^(1) = .01	.918
Civil status, *n* (%)			X^2^(4) = 10.81	.029
Never married	25 (50%)	14 (27%)		
Married	13 (26%)	16 (31%)		
Living together	3 (6%)	14 (27%)		
Divorced	8 (16%)	6 (12%)		
Widowed	1 (2%)	1 (2%)		
Work situation, *n* (%)			X^2^(6) = 27.39	<.001
Household	1 (2%)	1 (2%)		
School/education	4 (8%)	11 (21.5%)		
Regular work full-time	6 (12%)	22 (43%)		
Regular work part-time	13 (26%)	11 (21.5%)		
Structured work	10 (20%)	4 (8%)		
Non-structured activities	15 (30%)	1 (2%)		
Other	1 (2%)	1 (2%)		
Educational level, *n* (%)			X^2^(2) = 3.77	.152
Primary school	1 (2%)			
Secondary school	12 (24%)	6 (12%)		
Higher education	37 (74%)	45 (88%)		
Clinical variables				
ADOS-2 classification, *n*				
Autism	32			
Autism spectrum	18			
AQ score, mean (SD), range		9.4 (4.9), 0-25		
WAIS-IV subtests, mean (SD), range				
Matrix reasoning	11.0 (2.6), 6-18	10.9 (2.2), 5-15	F = .03	.874
Vocabulary	11.8 (2.9), 5-16	11.4 (3.0), 6-19	F = .40	.530
Estimated IQ, mean (SD), Range	110.1 (17.7), 79-147	108.5 (15.4), 73-141	F = .23	.636
DSM-IV axis I diagnosis *n*				
Depression current	3	0†	X^2^(1) = 3.15	.076
Depression lifetime	23	6	X^2^(1) = 14.46	<.001
Valid ESM beeps, mean (SD), range	79.8 (12.7), 49-103	75.8 (12.9), 32-97	F = 2.51	.116

^†^Current depression was an exclusion criterion in the control group; ASD, Autism Spectrum Disorder; ADOS-2, Autism Diagnostic Observation Schedule II; AQ, Autism Spectrum Quotient; IQ, intelligence quotient; WAIS-IV, Wechsler Adult Intelligence Scale - Fourth Edition; ESM, Experience Sampling Method.

### Procedure

This study was approved by the medical ethics committee of Maastricht University (NL51997.068.15) and was carried out in accordance with the Declaration of Helsinki (46). All participants were well informed about the study and gave written informed consent before the first appointment. During the first appointment, participants were screened for meeting the inclusion criteria and they filled in the CAPE. The ESM protocol was explained in a following session.

Daily life assessments were done with the ESM, delivered via the PsyMate™ application. Participants received an iPod or downloaded the app on their smartphone. During 10 days, 10 times a day, the application sent an alert at random moments between 07:30 and 22:30 h. Participants then answered questions about mood, social context, and activities, completing their reports within an allotment of 10 min after the signal. The questionnaire consisted of 7-point Likert scales to capture momentary experiences and categorical questions to capture context (e.g., social context, activities). Participants were encouraged to follow their daily routines. All participants were contacted by telephone after 2 days of sampling to ask if they experienced any problems concerning the protocol. It was also possible for them to contact the researchers, if they had questions or experienced problems with the ESM data collection. Exclusion from the analysis followed in case less than 30% valid reports were acquired (30 out of 100), as previous work has shown that these data are less reliable (47). However, none of the participants were excluded for this reason. After collecting the data, participants were invited for a debriefing session and their experiences were evaluated.

### Measures

#### CAPE—Lifetime Psychic Experiences

The CAPE is a reliable and valid retrospective self-report questionnaire to assess the frequency and distress of a set of different symptom dimensions of the extended psychosis phenotype. The questionnaire consists of 42 items, and the frequency score is measured on a 4-point scale: never (1), sometimes (2), often (3), and nearly always (4). Distress is measured on a 4-point scale: not distressed (1), a bit distressed (2), quite distressed (3), and very distressed (4). For both the frequency scales as well as the distress scales, items are arranged around three dimensions, i.e., positive psychotic experiences (20 items), as well as subclinical negative (14 items), and depressive symptoms (8 items). The internal consistency for this sample was determined by calculating Cronbach’s alpha. Excellent internal consistency was found for the frequency scale (.90) and good internal consistency for the distress scale (.80) in the ASD group. In the control group, a good internal consistency was found for the frequency scale (.83) and an excellent score for the distress scale (.93). The total CAPE scores as well as the three individual dimensions (i.e., positive, negative, and depressive symptoms) were used as outcome measures.

#### ESM—Momentary Psychotic Experiences

PE were operationalized with four questions (“I feel suspicious”, “I can’t get these thoughts out of my head”, “My thoughts are influenced by others”, and “I hear voices that others don’t”). These questions were scored on 7-point Likert scales (1 = not, 7 = very) and were combined into a mean momentary PE measure.

#### ESM—Momentary Stress

Stress was conceptualized as subjectively appraised stress after regular daily life encounters or activities. Three different stress measures were obtained: activity-related, event-related, and social stress. Activity-related stress was operationalized, starting with the question “What are you doing?”. Three items followed this question, i.e., “I would rather do something else”; “This is difficult for me”, and “I can do this well”, reverse coded. These questions were scored on 7-point Likert scales (1= not, 7 = very) and were combined into a mean activity-related stress variable. Event-related stress was based on the question “What was the most important event since the last beep?”. Participants subsequently scored how pleasant/unpleasant the event was on a bipolar scale (-3 very unpleasant, 0 neutral, +3 very pleasant). Positive events (scores 1, 2, and 3) were recorded to zero, and negative scores were reverse coded (i.e., higher ratings reflect more stress). Lastly, social stress was operationalized by asking participants if they were in the company of others or alone. If in the company of others, they were asked to rate the item “I would prefer to be alone” (1–7).

#### ESM—Negative Affect

NA was assessed at each beep with five adjectives (down, insecure, lonely, anxious, irritated) rated on 7-point Likert scales (1 = not, 7 = very). However, detailed factor analyses based on the present ESM data showed that the item ‘irritated’ also had high negative cross-loadings on a positive affect measure (based on the items relaxed, enthusiastic, satisfied, and cheerful). Therefore, the mean of the items “down”, “insecure”, “lonely”, and “anxious” was used as a measure of NA in the analyses.

### Statistical Analysis

All analyses were carried out in Stata version 13.1 (48).

#### CAPE—Lifetime Psychic Experiences

Eight regression analyses were performed to test for differences in frequency of lifetime psychic experiences and degree of distress between adults with ASD and controls. First, two regression analyses were computed with the total CAPE sum scores (on both the frequency and the distress scale) as the dependent variables. Group, sex, and their interaction were added as the independent variables. Second, six regression analyses were performed with the individual CAPE dimensions (positive, negative, and depressive symptoms, again on both the frequency and distress scale) as the dependent variables. Again, group, sex, and their interaction were added as the independent variables. Moreover, previous research showed that young adults are more prone to develop PE than middle-aged and older adults (49), and individuals with depression and lower educational achievement are more vulnerable to develop PE (19, 50, 51). Therefore, we used age, lifetime depression, and education level as covariates, because these variables may partially explain variance in overall and dimensional CAPE scores. Lastly, the predicted marginal means were estimated from these models. In case of a significant two-way interaction, we computed the pairwise differences in simple slopes between the four groups (i.e., males with ASD, females with ASD, control females, and control males). When only a significant main effect for group was found, we estimated the marginal means between ASD and controls.

#### ESM—Momentary Psychotic Experiences

ESM data have a multilevel structure. Therefore, two-level mixed-effects regression models (using the “mixed” command in Stata) were used to analyze data, with observations (level 1) nested within subjects (level 2). The independent variables, their interactions, and the covariates were entered into the models as fixed effects. Random intercepts and random slopes were added at the subject level, using an unstructured covariance matrix for the random effects. Models were fitted using restricted maximum likelihood estimation (REML). Fixed effects were tested *via* Wald-type tests with α=.05 (two-sided). As a first step, five multilevel models were fitted to test whether momentary PE, NA, and the three stress variables (independent variables) differed between groups (dependent variable: 0 = controls, 1 = ASD). Next, four models were fitted for activity-related stress, event-related stress, social stress, or NA as a continuous predictor and momentary PE as the outcome variable. Age, lifetime depression, and education level were added as covariates in all models as these might explain part of the variance in momentary PE, similar to the lifetime CAPE scores in the previous subsection. We added two-way (stress/NA x group, stress/NA x sex, group x sex) and three-way (stress/NA x group x sex) interactions to test whether associations between stress or NA and PE differed by group or sex. Based on each fitted model, we computed the slopes (of stress or NA on PE) for all four groups (i.e., males with ASD, females with ASD, control females, and control males) with corresponding 95% confidence intervals (CIs). Given that the current sample size was expected to yield limited power to investigate a three-way interaction, we collapsed these to appropriate marginal slopes if the three-way interaction was not significant. Thus, instead, the marginal slopes for the two-way interaction between stress or NA and group were reported. Lastly, we computed, only in case of a significant three-way interaction, the pairwise differences between the simple slopes to investigate the effects of group and sex on PE.

#### Sensitivity Analysis

To verify whether the results of the main analyses were robust, we performed a sensitivity analysis. First, we excluded the few participants diagnosed with depression (ASD N = 3, controls N = 0). Since depression is known to be associated with perceived stress (52), NA (53), and PE (54) it might explain some variance in the results. Second, the item “I can’t get these thoughts out of my head” was excluded from the repeated analyses since one may argue that this item is related to persistent thinking, a known feature in ASD (55). Thus, for the sensitivity analysis, momentary PE were operationalized as the total sum of the items: “I feel suspicious”, “My thoughts are influenced by others”, and “I hear voices that others don’t”.

## Results

### CAPE—Lifetime Psychic Experiences

#### CAPE—Overall Scores

There were no significant effects found in sex or group x sex interaction terms for the overall CAPE scores on frequency and distress (**Table 2**). Results showed significant group differences in overall CAPE scores. Moreover, the margins demonstrated distinctly higher levels of lifetime CAPE sum scores and accompanying distress in participants with ASD than controls. The estimated marginal means are summarized in **Table 3**.

**Table 2:** Regression estimates of group, sex, and their interaction associated with CAPE overall score and subscale scores.

	Obs	B	SE	P	95% CI
**Lifetime psychic experiences**					
*Total sum*	101				
Group		.30	.07	<.001	[.15,.45]
Sex		.01	.07	.923	[-.13,.14]
Group x sex		.15	.10	.117	[-.04,.35]
*Positive symptoms*	101				
Group		.09	.07	.180	[-.04,.22]
Sex		-.02	.06	.726	[-.14,.10]
Group x sex		.13	.09	.130	[-.04,.30]
*Negative symptoms*	101				
Group		.48	.11	<.001	[.26,.70]
Sex		.00	.10	.970	[-.20,.21]
Group x sex		.16	.15	.259	[-.12,.45]
*Depressive symptoms*	101				
Group		.52	.12	<.001	[.27,.77]
Sex		.08	.11	.481	[-.15,.31]
Group x sex		.19	.16	.243	[-.13,.51]
**Degree of distress**					
*Total sum*	100				
Group		.55	.12	<.001	[.31,.80]
Sex		.06	.11	.568	[-.16,.29]
Group x sex		.22	.16	.168	[-.09,.54]
*Positive symptoms*	89				
Group		.45	.20	.023	[.06,.85]
Sex		.09	.19	.629	[-.28,.46]
Group x sex		.31	.25	.217	[-.19,.81]
*Negative symptoms*	99				
Group		.54	.12	<.001	[.30,.78]
Sex		.16	.11	.144	[-.06,.38]
Group x sex		.10	.16	.513	[-.21,.41]
*Depressive symptoms*	100				
Group		.72	.17	<.001	[.39, 1.06]
Sex		-.04	.16	.813	[-.35,.27]
Group x sex		.34	.22	.136	[-.11,.78]

Obs, number of observations; B, standardized regression coefficient; SE, standard error; CI 95%, 95% confidence interval. All models control for age, lifetime depression (yes/no), and education level. CAPE, Community Assessment of Psychic Experiences.

**Table 3:** Estimated marginal means for the CAPE overall score and subscale scores, per group.

	ASD (N = 50)				Controls (N = 51)			
	Margin	SE	P	95% CI	Margin	SE	P	95% CI
**Frequency**								
Total	1.73	.04	<.001	[1.66, 1.80]	1.36	.04	<.001	[1.29, 1.43]
Positive symptoms	1.29	.03	<.001	[1.23, 1.36]	1.14	.03	<.001	[1.08, 1.21]
Negative symptoms	2.11	.05	<.001	[2.00, 2.22]	1.55	.05	<.001	[1.45, 1.66]
Depressive symptoms	2.16	.06	<.001	[2.04, 2.28]	1.55	.06	<.001	[1.43, 1.67]
**Distress**								
Total	2.27	.06	<.001	[2.15, 2.38]	1.60	.06	<.001	[1.49, 1.72]
Positive symptoms	2.01	.09	<.001	[1.84, 2.18]	1.40	.10	<.001	[1.20, 1.59]
Negative symptoms	2.17	.06	<.001	[2.05, 2.28]	1.58	.06	<.001	[1.46, 1.69]
Depressive symptoms	2.72	.08	<.001	[2.56, 2.89]	1.83	.08	<.001	[1.67, 2.00]

SE, standard error; 95% CI, 95% confidence interval; ASD, Autism Spectrum Disorder; CAPE, Community Assessment of Psychic Experiences.

#### CAPE—Symptom Dimensions

None of the individual CAPE symptom dimensions were significantly associated with the interaction between group and sex, and no significant effects were found for sex (all P >.05) (**Table 2**). The results showed significant group differences for all three symptom dimensions of frequency and distress (see **Tables 2** and **3**) except for the positive symptom frequency scale. Thus, adults with ASD reported higher levels of negative and depressive symptoms on the CAPE frequency scale and higher levels of accompanying distress on all three symptom dimensions.

### ESM—Momentary Psychotic Experiences

Higher levels of momentary PE were found in adults with ASD relative to controls (**Table 4**).

**Table 4:** Multilevel regression estimates of the ESM variables between groups.

	Obs	B	SE	P	95% CI
Negative affect	7846	.83	.14	<.001	[.56,.1.10]
Activity-related stress	7844	.61	.14	<.001	[.34,.88]
Event-related stress	7836	.09	.04	.028	[.01,.17]
Social stress	4696	1.21	.20	<.001	[.82, 1.60]
Psychotic experiences	7845	.49	.11	<.001	[.28,.70]

Obs, number of observations; B, standardized regression coefficient; SE, standard error; CI 95%, 95% confidence interval; ESM, Experience Sampling Method.

### ESM—Momentary Stress and Negative Affect

The ASD group reported significantly higher levels of NA, activity-related, event-related, and social stress than controls (**Table 4**). Note that the number of observations of the social stress variable was lower than for the other predictors because social stress was only measured in situations where participants reported being in the company of others.

### ESM—the Impact of Daily Life Stressors on Momentary Psychotic Experiences

#### Activity-Related Stress

The interaction between activity-related stress, group, and sex in the model of momentary PE was not significant; neither was the two-way interaction between group and activity-related stress. There were significant main effects of both activity-related stress and group (**Table 5**).

**Table 5:** Multilevel regression estimates of stress, group, sex, and their interactions in the model of momentary psychotic experiences.

	Obs	B	SE	P	95% CI
1. Activity-related stress	7843	.04	.02	.032	[.00,.08]
Group		.26	.13	.046	[.00,.51]
Group x activity-related stress		.05	.03	.063	[-.00,.10]
Sex		.12	.12	.320	[-.12,.35]
Sex x activity-related stress		-.01	.03	.773	[-.06,.05]
Sex x group		-.08	.17	.629	[-.42,.25]
Group x sex x activity-related Stress		.03	.04	.430	[-.04,.11]
2. Event-related stress	7835	.04	.03	.195	[-.02,.10]
Group		.32	.16	.041	[.01,.63]
Group x event-related stress		.11	.04	.006	[.03,.19]
Sex		.11	.15	.468	[-.18,.39]
Sex x event-related stress		.02	.04	.558	[-.06,.11]
Sex x group		.05	.21	.797	[-.35,.46]
Group x sex x event-related stress		-.03	.06	.599	[-.14,.08]
3. Social stress	4695	.02	.02	.298	[-.02,.07]
Group		.28	.14	.049	[.00,.56]
Group x social stress		.02	.03	.404	[-.03,.08]
Sex		.10	.13	.470	[-.16,.35]
Sex x social stress		.04	.03	.223	[-.02,.10]
Sex x group		.01	.19	.942	[-.36,.39]
Group x sex x social stress		-.02	.04	.711	[-.10,.06]
4. NA	7842	.21	.05	<.001	[.11,.31]
Group		.05	.11	.607	[-.15,.26]
Group x NA		.10	.07	.121	[-.03,.23]
Sex		.11	.10	.262	[-.08,.30]
Sex x NA		.01	.07	.914	[-.13,.14]
Sex x group		.03	.14	.840	[-.25,.30]
Group x sex x NA		.01	.09	.933	[-.17,.19]

Obs, number of observations; B, standardized regression coefficient; SE, standard error; CI 95%, 95% confidence interval; NA, negative affect. The dependent variable in all models is psychotic experiences. All models control for age, lifetime depression, and education level.

#### Event-Related Stress

**Figure 1:**
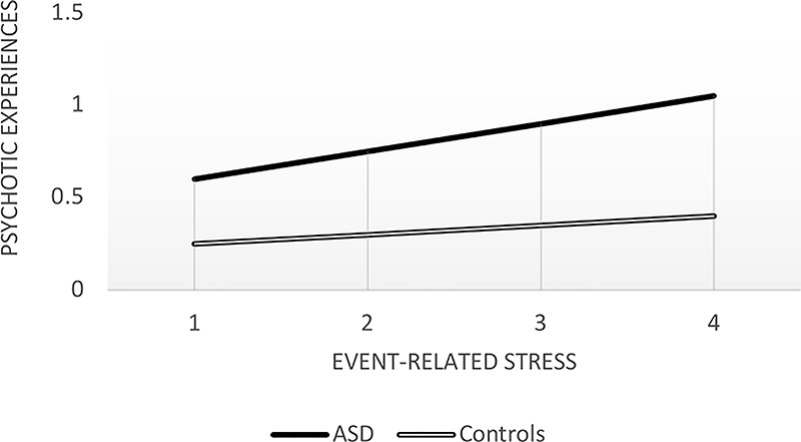
Associations between event-related stress scores and psychotic experiences. ASD, Autism spectrum disorder.

The analyses showed no significant three-way interaction. As shown in **Table 5**, a significant two-way interaction was found between group and event-related stress in the model of momentary PE. The results of the simple slopes showed a stronger association between event-related stress and PE in the ASD group (B = .15, S.E. = .02, P <.001, 95% CI [.11,.19]) than in controls (B = .05, S.E. = .02, P = .016, 95% CI [.01/.09]) (see **Figure 1**).

#### Social Stress

No significant interaction was found between group, sex, and social stress nor between group and social stress in the model of momentary PE. Results demonstrated a trend-significant main effect for group.

### ESM—the Impact of Negative Affect on Momentary Psychotic Experiences

The results showed a non-significant three-way interaction (group x sex x NA), and two-way interaction (group x NA) in the model of momentary PE. The analyses showed a significant effect of NA on momentary PE (**Table 5**).

### Sensitivity Analysis

Additional analyses were carried out, excluding participants diagnosed with depression from the sample (ASD: N = 3, controls: N = 0), and with momentary PE as the total sum of three items instead of four (the item “I can’t get these thoughts out of my head” was excluded). All analyses were repeated within the new sample (ASD N = 47, controls N = 51). The results remained similar for all analyses except one: a significant two-way interaction was found between activity-related stress and group on momentary PE (B = .05, S.E. = .02, P = .019, 95% CI [.01,.08]). Marginal effects of the interaction term showed that activity-related stress was significantly associated with momentary PE in the ASD group (B = .07, S.E. = .01, P <.001, 95% CI [.05,.08]) but not in the control group (B = .02, S.E. = .01, P = .094, 95% CI [-.00,.04]). All results are presented in **Supplementary Tables S1** and **S2**.

## Discussion

The current study aimed at acquiring more insight into (subclinical) psychotic symptom expression and potential contributing risk factors in adults with ASD. Participants with ASD reported significantly higher lifetime CAPE sum scores (reflecting the extended psychosis phenotype), as well as higher lifetime subclinical negative and depressive symptom scores, all accompanied with higher levels of distress than controls. Although no significant group differences were found in lifetime CAPE PE scores, the ASD group reported more accompanying distress than controls. Adults with ASD reported more ESM momentary PE than controls and event-related stress was associated with increased momentary PE in adults with ASD. There was no moderating effect of group on the associations between either activity-related stress, social stress, or NA, and the outcome variable momentary PE. Overall, no significant differences between male and female outcomes were found.

### CAPE—Lifetime Psychic Experiences

Adults with ASD reported significantly more lifetime experiences related to the extended psychosis phenotype, including higher levels of distress. Analyses of the sub-dimensions showed that adults with ASD reported higher levels of negative and depressive symptoms compared to controls, but not higher levels of PE. Although the latter finding differs from previous literature, current results are in line with studies that found a stronger association between autistic features and negative, rather than positive, symptoms (56, 57). Moreover, this is the first ASD study to investigate distress related to symptoms of the extended psychosis phenotype. Higher levels of distress were found in the ASD group compared with controls, for the total scale and all three sub-dimensions. Thus, even though no evidence was found that individuals with ASD have more lifetime PE, they experienced more distress from those experiences than controls. These findings are (clinically) informative since previous studies showed that distress related to PE, rather than frequency of PE, is associated with a higher risk of developing clinical need (58–61). The increased frequency and distress levels of negative and depressive symptoms also point out that clinicians and caregivers should be alerted for a transdiagnostic approach in the mental health care of individuals with ASD, encompassing support and treatment interventions for these extended psychosis phenotype features. Moreover, higher levels of distress related to lifetime experiences may suggest that stress sensitivity plays a role in the emergence of PE (62, 63) in adults with ASD. More specifically, previous research has demonstrated that early trauma and adverse life events can result in altered stress sensitivity, which in turn may lead to a higher frequency and intensity of PE later in life. This pathway has been described as the “affective pathway towards psychosis” (34, 62). An increased stress sensitivity in adults with ASD may be due to a higher vulnerability for childhood adversities, e.g., family and neighborhood adversities (64), and peer victimization (65, 66).

No sex differences were found with respect to lifetime psychic experiences. Given that there is no previous research available, it is not possible to make direct comparisons. However, our results seem to be in contrast with the replicated finding from general population studies that males experience more negative symptoms while females experience more positive symptoms (67, 68). Although there is one general population study that showed higher levels of the CAPE total frequency scale in females (69), this study did not provide data on the subscales. The current results suggest that negative and depressive symptoms are related to ASD in general instead of being sex-dependent. Still, since this is the first study investigating lifetime experiences in adult males and females with ASD, more studies are warranted to further examine this topic. Future studies should aim for larger sample sizes since the current sample was relatively small to investigate sex differences. Furthermore, previous studies examining sex differences in co-occurring symptoms (e.g., anxiety) found significant differences in children and adolescents with ASD (70–72), but not in adults (73–75). Therefore, the results of the present study may not be illustrative of the complete lifespan.

### ESM—Momentary Psychotic Experiences

#### Levels of Momentary Psychotic Experiences

Despite the absence of group differences in frequency levels of CAPE PE, higher levels of ESM momentary PE were demonstrated in ASD. This may indicate that real-time, real-world, daily life monitoring can signal (small changes in) PE, whereas a retrospective instrument may lack the sensitivity to do so. This urges the need for the combination of well-validated (retrospective and EMA) instruments to investigate transdiagnostic phenomenological features in ASD, as the different approaches may be (partly) complementary. Of note, concerning affect, the two instruments yielded overlapping results. As no questions on negative symptoms were included in the ESM questionnaire for this study, this may be a consideration for future research on ASD. In summary, the present study showed the feasibility and relevance of studying momentary PE in a naturalistic environment.

#### The Impact of Daily Stressors on Momentary Psychotic Experiences

The ASD group showed higher PE levels in association with event-related stress than the control group. Another paper on this sample demonstrated that adults with ASD report higher levels of NA associated with event-related stress, i.e., increased stress-reactivity (submitted for publication). Findings seem to concur with research reporting on unpleasant events as an important stressor in individuals with ASD (76, 77). The absence of a moderating effect of sex could be related to a lack of power. Still, it may also indicate that an increased psychotic reactivity associated with event-related stress is characteristic of ASD in general. Group and sex had no significant effect on the association between activity-related stress and momentary PE. Although, the interaction between group and activity-related stress did reach significance in the sensitivity analysis. No significant moderation effects of group and sex on social stress in the association with momentary PE were found. This was unexpected, especially since problems in social functioning and communication have been found in ASD as well as in individuals who are at clinical high risk for psychosis and individuals with a first psychotic episode (78). The results may comply with a longitudinal study of Bevan Jones et al. (10), which observed that maternal concerns about social interaction in childhood were not significantly associated with increased PE in adolescence. The present findings show that even though adults with ASD reported an increased desire to be alone when in the company of others, momentary PE levels associated with social stress were comparable in both groups. A possible explanation for these findings may be that adults with ASD do experience benefits of social contact (79, 80). It may be that the presence of social support provides a feeling of safety and improves quality of life (81). Thus social support may be a protective factor for momentary PE in ASD, in agreement with the results of a recent longitudinal cohort study in the general population (82).

#### The Impact of Negative Affect on Momentary Psychotic Experiences

Results showed a significant association between NA and momentary PE but no significant effect of sex and group. Despite the lack of significant group differences, these findings are in line with research that demonstrated an association between NA and momentary PE (34, 36). Moreover, although adults with ASD reported significantly higher levels of NA than controls, the current findings implicate that NA is not a specific risk factor for momentary PE. Nevertheless, in line with the affective pathway to psychosis as described in subsection *CAPE—Lifetime Psychic Experiences*, research has shown that increased NA in response to daily stress is associated with higher levels of lifetime CAPE scores in the general population (83). Therefore, it may be suggested that NA should not be viewed as a separate risk factor, but may lie on the causal pathway between stress and momentary PE.

### Clinical Implications

Present findings highlight the critical role of stress with the emergence of PE in ASD. Results demonstrated that adults with ASD not only experience higher levels of distress in response to (lifetime) PE, but also that stressful events in daily life may increase momentary PE. This may lead to a vicious cycle where adults with ASD may feel distressed by their PE, which, in turn, increases the frequency and intensity of PE. Stress prevention may be one way to disrupt this cycle. Although research on treatment interventions in adults with ASD is limited, some studies demonstrated that cognitive-behavioral therapy (84), acceptance and commitment therapy (85), and dog-assisted therapy (86) led to a significant stress reduction in this population.

### Strengths and Limitations

Previous research mainly investigated PE in the ASD population using standard clinical measures. We have tried to bridge the gap in the present literature by examining both self-reported lifetime experiences and momentary assessment of PE in a naturalistic environment. Another strength is that this study included an equal number of males and females, while most research in ASD is focused on male children and adolescents. Furthermore, an ASD group with minimal treatment history was included, and therefore it was possible to examine psychotic reactivity minimally influenced by prior treatment. Although we included a relatively large sample and a sufficient number of self-reports, a lack of power may have affected the three-way interactions. Furthermore, it may be questioned whether all the items used to investigate momentary PE were suitable for the ASD group. A previous study (87) from our department showed, however, that these momentary PE were strongly associated with the positive symptom items of the Positive and Negative Symptom Scale (PANSS) (88) in patients with a psychotic disorder. Lastly, a high functioning group was included, and therefore results may not be generalized to the whole ASD spectrum.

### Conclusion

Current results underline that adults with ASD are more prone to encounter lifetime extended psychosis phenotype features, i.e., subclinical negative and depressive symptoms, accompanied with more distress. Even though no group differences were found in the frequency of lifetime PE, these symptoms were accompanied with greater distress in ASD. Results showed higher levels of momentary PE in adults with ASD compared to controls. Furthermore, event-related stress was associated with increased levels of momentary PE, indicating increased psychotic reactivity, in participants with ASD. No significant differences between males and females were found.

## Data Availability Statement

The datasets presented in this article are not readily available because of patient confidentiality and participant privacy. Requests to access the datasets should be directed to MM (m.marcelis@maastrichtuniversity).

## Ethics Statement

The studies involving human participants were reviewed and approved by Medisch-ethische toetsingscommissie azM/UM, Maastricht, the Netherlands. The patients/participants provided their written informed consent to participate in this study.

## Author Contributions

All authors contributed to conception and design of the study. KL organized the database, performed the statistical analysis, and wrote the first draft of the manuscript. CS, TA, and MM critically reviewed the manuscript. All authors contributed to the article and approved the submitted version.

## Funding

This work was supported by Geestelijke Gezondheidszorg Eindhoven (GGzE) and Maastricht University. The open access publication fees were supported by Maastricht University.

## Conflict of Interest

The authors declare that the research was conducted in the absence of any commercial or financial relationships that could be construed as a potential conflict of interest.
