# Supplementary material for: Lifetime and Momentary Psychotic Experiences in Adult Males and Females With an Autism Spectrum Disorder
Source: Front Psychiatry. 2020 Aug 3;11:766. doi: 10.3389/fpsyt.2020.00766 (PMC7416642; doi:10.3389/fpsyt.2020.00766)
Supplement: Supplementary file 1 [file Table_1.docx]

***Supplementary Material: Results Sensitivity Analysis***

**Table S1. Regression estimates of group, sex and their interaction associated with CAPE overall score and subscale scores**

|  | **Obs** | **B** | **SE** | **P** | **95% CI** |
| --- | --- | --- | --- | --- | --- |
|  |  |  |  |  |  |
| **Lifetime psychic experiences** |  |  |  |  |  |
| *Total sum* | 98 |  |  |  |  |
| Group |  | .31 | .06 | < .001 | [.18, .43] |
| Sex |  | .02 | .06 | .759 | [-.09, .13] |
| Group x sex |  | .10 | .08 | .199 | [-.06, .26] |
| *Positive symptoms* | 98 |  |  |  |  |
| Group |  | .09 | .05 | .049 | [.00, .19] |
| Sex |  | -.01 | .04 | .815 | [-.10, .08] |
| Group x sex |  | .08 | .06 | .224 | [-.05, .20] |
| *Negative symptoms* | 98 |  |  |  |  |
| Group |  | .50 | .10 | < .001 | [.29, .70] |
| Sex |  | .01 | .09 | .883 | [-.17, .20] |
| Group x sex |  | .10 | .14 | .477 | [-.17, .37] |
| *Depressive symptoms* | 98 |  |  |  |  |
| Group |  | .50 | .12 | < .001 | [.26, .74] |
| Sex |  | .09 | .11 | .407 | [-.13, .31] |
| Group x sex |  | .19 | .16 | .241 | [-.13, .50] |
|  |  |  |  |  |  |
| **Degree of distress** |  |  |  |  |  |
| *Total sum* | 97 |  |  |  |  |
| Group |  | .55 | .12 | < .001 | [.31, .79] |
| Sex |  | .07 | .11 | .527 | [-.15, .29] |
| Group x sex |  | .20 | .16 | .205 | [-.11, .51] |
| *Positive symptoms* | 86 |  |  |  |  |
| Group |  | .46 | .20 | .021 | [.07, .85] |
| Sex |  | .10 | .18 | .599 | [-.27, .46] |
| Group x sex |  | .28 | .25 | .273 | [-.22, .77] |
| *Negative symptoms* | 96 |  |  |  |  |
| Group |  | .53 | .12 | < .001 | [.30, .76] |
| Sex |  | .17 | .11 | .111 | [-.04, .38] |
| Group x sex |  | .08 | .15 | .613 | [-.23, .38] |
| *Depressive symptoms* | 97 |  |  |  |  |
| Group |  | .70 | .17 | < .001 | [.36, 1.04] |
| Sex |  | -.03 | .16 | .840 | [-.34, .28] |
| Group x sex |  | .36 | .23 | .117 | [-.09, .81] |

Obs, number of observations; B, standardized regression coefficient; SE, standard error; CI 95%, 95% confidence interval. All models control for age, lifetime depression (yes/no), and education level. CAPE, Community Assessment of Psychic Experiences

**Table S2. Multilevel regression estimates of stress, group, sex and their interactions in the model of momentary psychotic experiences**

|  | **Obs** | **B** | **SE** | **P** | **95% CI** |
| --- | --- | --- | --- | --- | --- |
|  |  |  |  |  |  |
| 1. Activity-related stress | 7630 | .02 | .01 | .176 | [-.01, .05] |
| Group |  | .14 | .10 | .166 | [-.06, .35] |
| Group x activity-related stress |  | .05 | .02 | .019 | [.01, .08] |
| Sex |  | .06 | .09 | .531 | [-.13, .24] |
| Sex x activity-related stress |  | -.00 | .02 | .812 | [-.04, .03] |
| Sex x group |  | -.14 | .14 | .318 | [-.41, .13] |
| Group x sex x activity-related stress |  | .01 | .03 | .837 | [-.05, .06] |
|  |  |  |  |  |  |
| 2. Event-related stress | 7621 | .02 | .03 | .510 | [-.03, .07] |
| Group |  | .22 | .11 | .055 | [-.00, .44] |
| Group x event-related stress |  | .11 | .04 | .004 | [.03, .18] |
| Sex |  | .06 | .11 | .539 | [-.14, .27] |
| Sex x event-related stress |  | .02 | .04 | .607 | [-.05, .09] |
| Sex x group |  | -.10 | .15 | .529 | [-.39, .20] |
| Group x sex x event-related stress |  | -.02 | .05 | .637 | [-.12, .08] |
|  |  |  |  |  |  |
| 3. Social stress | 4580 | .00 | .02 | .843 | [-.03, .04] |
| Group |  | .17 | .11 | .126 | [-.05, .39] |
| Group x social stress |  | .04 | .03 | .155 | [-.01, .09] |
| Sex |  | .04 | .10 | .700 | [-.16, .24] |
| Sex x social stress |  | .04 | .03 | .166 | [-.02, .09] |
| Sex x group |  | -.08 | .15 | .612 | [-.37, .22] |
| Group x sex x social stress |  | -.03 | .04 | .409 | [-.10, .04] |
|  |  |  |  |  |  |
| 4. NA | 7628 | .12 | .05 | .014 | [.02, .21] |
| Group |  | .03 | .08 | .698 | [-.12, .18] |
| Group x NA |  | .09 | .06 | .177 | [-.04, .21] |
| Sex |  | .05 | .07 | .480 | [-.09, .18] |
| Sex x NA |  | .02 | .06 | .760 | [-.11, .15] |
| Sex x group |  | -.06 | .10 | .545 | [-.26, .14] |
| Group x sex x NA |  | -.00 | .09 | .963 | [-.18, .17] |
|  |  |  |  |  |  |

Obs, number of observations; B, standardized regression coefficient; SE, standard error; CI 95%, 95% confidence interval; NA, negative affect. Dependent variable in all models is psychotic experiences. All models control for age, lifetime depression, and education level.
